# Post-Stroke Delusional Misidentification Syndrome: A Case Study

**DOI:** 10.1192/bjo.2026.11890

**Published:** 2026-06-30

**Authors:** Amr Romeh, Linda Saji

**Affiliations:** Cardiff and Vale University Health Board, Cardiff, United Kingdom

## Abstract

**Aims:**

Delusional Misidentification Syndromes represent disorders that are neuropsychiatric in nature. They include four main disorders: Capgras Syndrome, Frégoli Syndrome, Intermetamorphosis and Syndrome of Subjective Doubles. These syndromes are more noted in context of Schizophrenia but they can be encountered in other psychiatric disorders. Strokes and head traumas have also been implicated as contributing factors/causes for the development of these syndromes. Mainstay of treatment is antipsychotic medications.

**Methods:**

75-year-old gentleman with no past psychiatric history, had a stroke that affected “posterior left insula and left temporal operculum”.A “right frontal and right parietal infarcts” were established on his head scan at the time. The right frontal infarct was present on an MRI three months prior but the right parietal one was not. Following the stroke, he presented with receptive and expressive dysphasia but no motor symptoms. There were elements of mild cognitive impairment but the main symptoms align with Delusional Misidentification Syndrome, subtype Syndrome of Subjective Doubles. He became convinced that there are people that look exactly like him, including “a brother who had facial surgery” to look like him. Of note, the patient does not have brothers. Another “double” is “someone who lives in a care home and is in a vegetative state”. Interestingly, the patient was residing in a care home for few months.

**Results:**

Timeline of events reflects that the patient lived alone independently in his home till he had a stroke and was admitted to hospital. Post-stroke rehabilitation efforts were not successful and he was discharged to a care home. After a while, the care home struggled to meet his needs due to emergence of frequent suspiciousness that led to aggressive episodes. He was later detained to a psychiatric ward for a period of assessment and treatment. The patient’s function and quality of life was clearly affected as a consequence of the stroke. Due to the patient’s dysphasia, it was difficult to understand his psychiatric symptoms. It took some time until his symptoms were pieced together indicating Delusional Misidentification Syndrome. Improvement noted once Olanzapine was started and dose increased up to 10mg.

**Conclusion:**

Delusional Misidentification Syndromes are rare neuropsychiatric disorders that require treatment with antipsychotic medications. They also have some organic basis and some links to strokes and traumatic brain injuries. This case is vital in understanding the link between neurology and psychiatry and offers further notions to clinicians to reflect more on the organic nature of mental disorders.

